# Gold Compounds and the Anticancer Immune Response

**DOI:** 10.3389/fphar.2021.739481

**Published:** 2021-09-13

**Authors:** Ling Zhou, Huiguo Liu, Kui Liu, Shuang Wei

**Affiliations:** Department of Respiratory and Critical Care Medicine, Tongji Hospital, Tongji Medical College Huazhong University of Science and Technology, Wuhan, China

**Keywords:** gold compounds, anticancer drug, immune response, nanoparticles of gold compounds, immunotherapeutic

## Abstract

Gold compounds are not only well-explored for cytotoxic effects on tumors, but are also known to interact with the cancer immune system. The immune system deploys innate and adaptive mechanisms to protect against pathogens and prevent malignant transformation. The combined action of gold compounds with the activated immune system has shown promising results in cancer therapy through *in vivo* and *in vitro* experiments. Gold compounds are known to induce innate immune responses; however, these responses may contribute to adaptive immune responses. Gold compounds play the role of a major hapten that acts synergistically in innate immunity. Gold compounds support cancer cell antigenicity and promote anti-tumor immune response by inducing the release of CRT, ATP, HMGB1, HSP, and NKG2D to enhance immunogenicity. Gold compounds affect various immune cells (including suppressor regulatory T cells), inhibit myeloid derived suppressor cells, and enhance the function and number of dendritic cells. Gold nanoparticles (AuNPs) have potential for improving the effect of immunotherapy and reducing the toxicity and side effects of the treatment process. Thus, AuNPs provide an ideal opportunity for exploring the combination of anticancer gold compounds and immunotherapeutic interventions.

## Introduction

**FIGURE 1:**
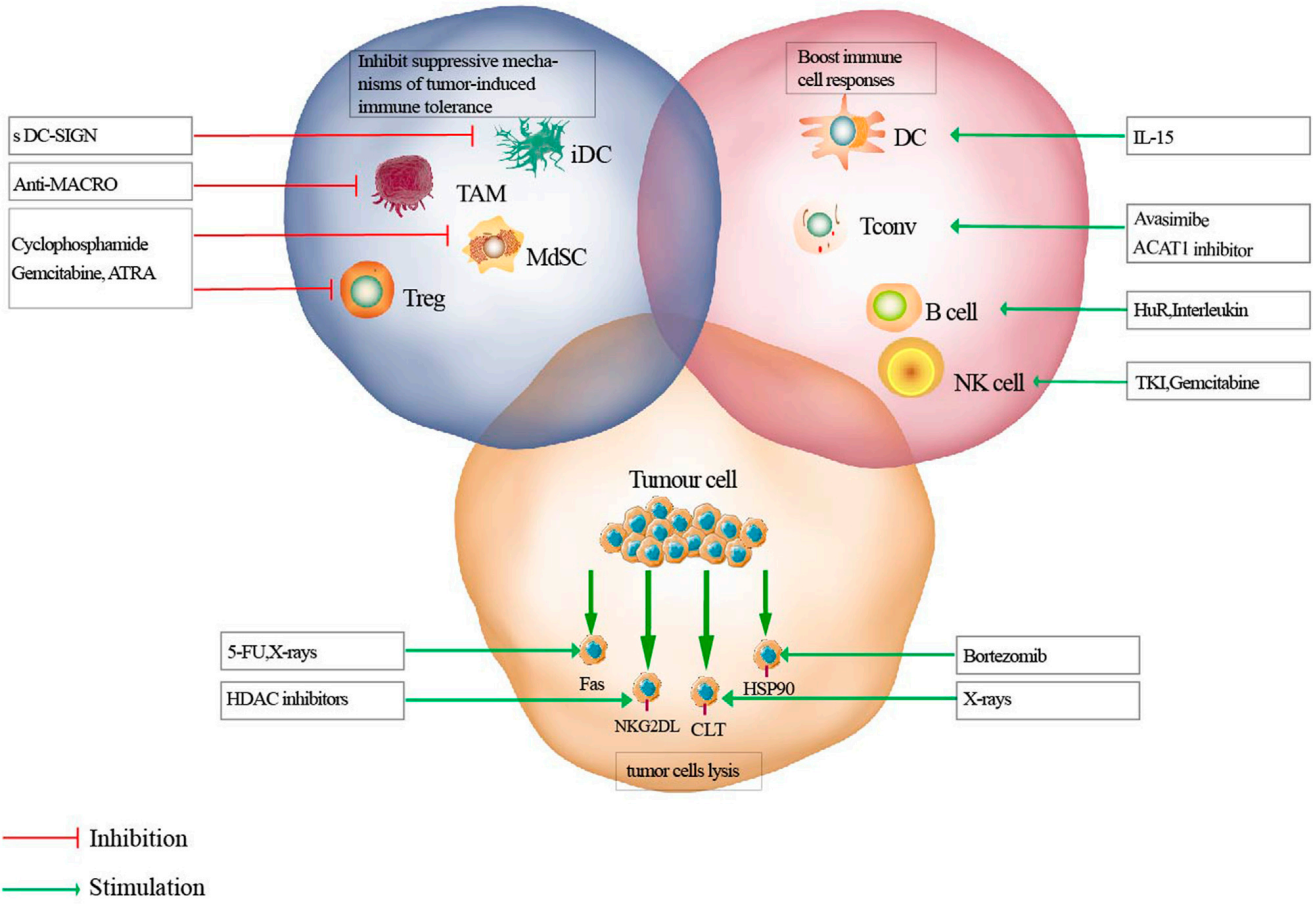
Mechanisms of effective anti-tumor therapies on immune responses. Antitumor therapeutics can eliminate immunosuppressive cells (blue circle), activate immune effectors (pink circle), or sensitize tumor cells to lysis (yellow circle). iDC, immature dendritic cell; TAM, tumor associated macrophage; MdSC, myeloid derived suppressor cells; Tconv, conventional T cell; ATRA, all-trans-retinoic acid; TKI, tyrosine kinase inhibitors.

In recent years, accumulating evidence has suggested that all types of cancer therapies, including surgery, radiation, chemotherapy. and targeted drugs, (except for immunotherapy), might result in opposing effects on systemic and cancer-associated immunological parameters. It is well-established that chemotherapy may result in immunosuppression (Brown et al., 2018). However, an appropriate combination of immunotherapy and cytotoxic chemotherapy may exhibit a highly synergistic anticancer activity. Tumorigenesis is the result of gene mutations, abnormal expression, or deletions under the influence of genetic and environmental factors, which eventually leads to abnormal cell proliferation. A non-compromised immune system can detect, recognize, and eradicate tumor cells. However, the interaction between tumor cells and the immune system is regulated by a large number of immune activator/inhibitor molecules. Due to genetic instability, tumors show a high degree of heterogeneity, which is a characteristic of malignant tumors. Heterogeneity results in multiple interactions between the tumor and the host immune response, affecting immunotherapy. Consequently, an anti-tumor immune response is produced to inhibit the occurrence and development of tumors. The main anticancer reactions induced by anticancer drugs include elimination of immunosuppressive cells, activation of immune effectors, and sensitization of tumor cells to lysis. Ideally, appropriate anticancer therapeutic agents should achieve all three of these beneficial effects (Figure 1). However, due to the immunosuppressive factors in cancer patients, the effect of immunotherapy alone remains insufficient. Consequently, decreasing tumor immunosuppression and improving anti-tumor immune responses have become the major research focus areas in the development of effective anticancer therapies. A class of cytotoxic agents that show potential for novel combination strategies with immunotherapies are anticancer metal drugs, including gold compounds. This review implicates a complex interplay between novel anticancer gold compounds and the anticancer immune response, both directly affecting immune effectors and cancer cell immune recognition.

## Anticancer Gold Compounds and the Immune System

### Immune Response Contributes to the Anticancer Activity of Gold Compounds

Gold compound-based anticancer therapeutics induce immune-potentiating effects that are predominantly initiated by damage to the cancer cells, signals released by drug-exposed tumor tissues, and the recruitment of effector immune cells into the cancer lesions, which counteract the immune-resistant milieu of the tumor microenvironment (TME) (Rivera Vargas and Apetoh, 2017). Gold (I) compounds not only act on tumor cells and immune cells directly, but also affect the expression of cell adhesion molecules on endothelial cells (Eisler, 2003). Chemotherapy has been shown to increase the risk of secondary infections via myelosuppression and lymphocytopenia, indicating that it may lead to immune suppression (van der Most et al., 2005). However, an appropriate combination of cytotoxic chemotherapy and immunotherapy may exert a highly synergistic anticancer activity (Gandhi et al., 2018). Chemotherapy can potentially affect certain immune cells that may result in enhanced anticancer effects and reverse “immune evasion” of cancer cells. Cytotoxic chemotherapy and anticancer immune responses are multifaceted and complex events (Brown et al., 2018; Wu and Waxman, 2018). Additionally, dose, schedule, and interconnection in treatment modalities also affect prognosis. Recent advances in the field suggest that anticancer metal drugs in combination with immunotherapies might become novel focus areas for effective anticancer therapeutic interventions (Dilruba and Kalayda, 2016).

### Efficacy of Gold Compounds in Cancer Therapy

Gold (Au) has been used in medicinal preparations since ancient times, with the earliest records by the Chinese and Egyptians dating back to ∼2500 BC (Faa et al., 2018). Metal drugs, including gold (Au) and arsenic (As) compounds, are some of the oldest remedies employed by humans ranging from the ancient Chinese to modern societies to fight a broad array of diseases, including cancer (Nicolis et al., 2009; Nobili et al., 2010). The synthesis and application of novel anticancer drugs remains an active field in inorganic medicinal chemistry (Allardyce and Dyson, 2016; Johnstone et al., 2016). Although only few metal drugs have been approved for clinical use in oncology, metal drugs still play a key role in therapeutic interventions for many cancers (Muggia et al., 2015). In addition to some clinically approved platinum drugs, several other metal drugs such as ruthenium (Ru), titanium (Ti), gallium (Ga), and gold compounds, have entered the stage of clinical evaluation (Chitambar, 2017; Lazarevic et al., 2017; Liang et al., 2017). The development of new metal-based cancer therapeutics apart from platinum drugs is a major goal of modern medical and bio-organometallic chemistry research. Gold compounds such as RANCE-1 are worth investigating for their role in anticancer immune surveillance (Madeira et al., 2012). Gold complexes show great potential for entering clinical trials because certain gold complexes are highly cytotoxic to solid cancerous tumors *in vitro* and *in vivo*, while causing minimal systemic toxicity. Accordingly, these complexes have become the subject of increased anticancer research in recent times.

**FIGURE 2:**
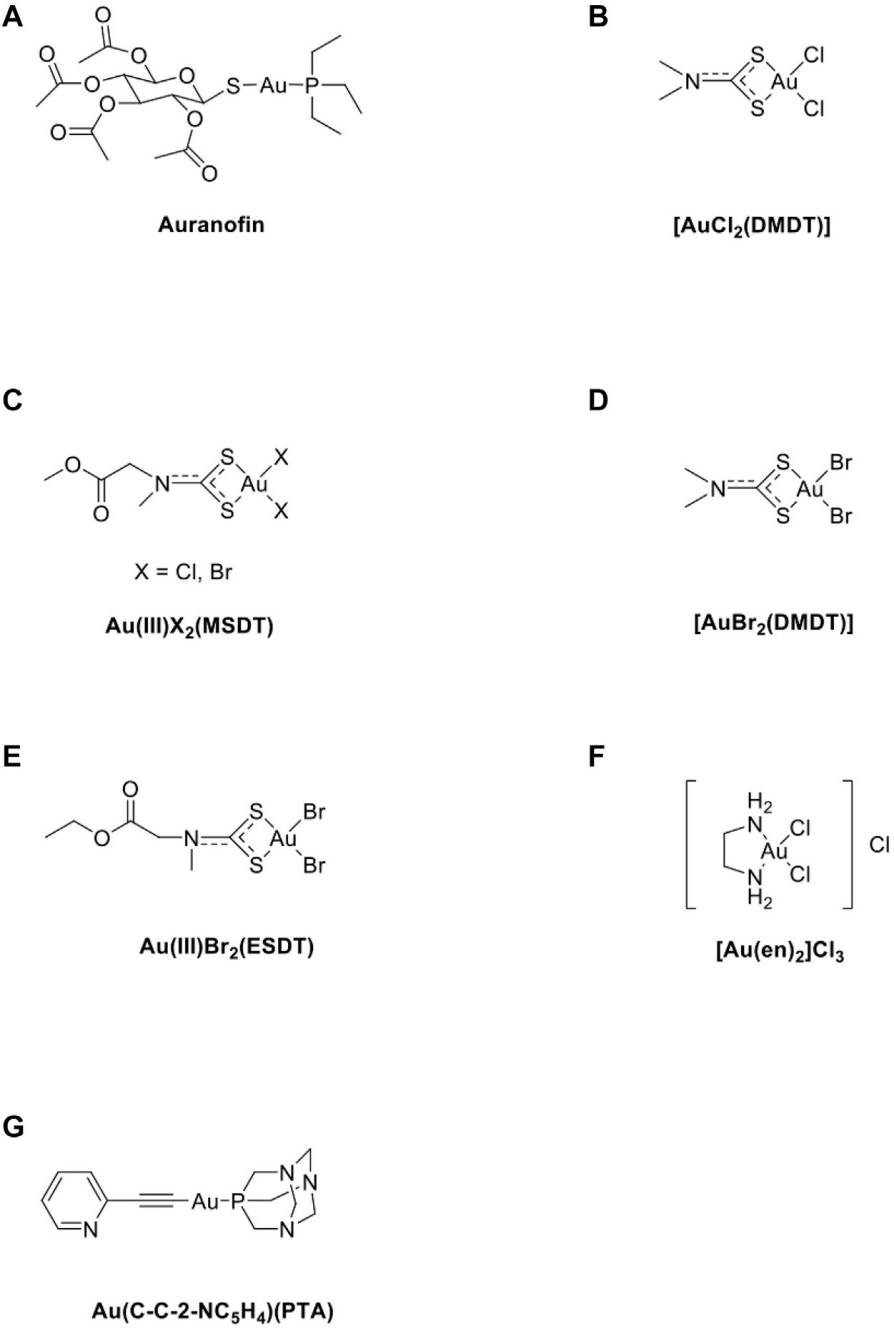
The chemical structure of several common gold compounds. **(A)** Auranofin; **(B)** Au^III^Cl_2_(DMDT); **(C)** AuX_2_(MSDT) X=Cl, Br; **(D)** Au(III)Br_2_(DMDT); **(E)** Au(III)Br2(ESDT); **(F)** [Au(en)_2_]Cl_3_; **(G)** Au(C-C-2-NC_5_H_4_)(PTA).

Despite massive chemical synthesis, preclinical evaluation approaches, and virtual generation of thousands of metal complexes with anticancer activity, only a handful of complexes are clinically approved. This challenging task can be successful only if underpinned by enhanced knowledge on the complex effects of metal-based chemotherapy on the host’s immune system. The elemental forms of gold, principally metallic and colloidal gold, are stable. Au(0), Au(I), and Au(III) are the most important oxidation states for gold (Nobili et al., 2010). Au(I) complexes are thermodynamically more stable than Au(III) because Au(III) is more reactive than Au(I) which has been suggested to be responsible for its high toxicity and adverse effects (Faa et al., 2018). Auranofin (Figure 2A) is a gold compound that can induce ROS and apoptosis in cancer cells via the Au(I)/Au(III) redox system (Hou et al., 2018). Au(III)-methylsarcosinedithiocarbamate (MSDT) complexes are significantly more active than both cisplatin and their platinum(II) and palladium(II) counterparts under the same experimental conditions and induce apoptosis, especially in HL60 cells (Giovagnini et al., 2005). Additionally, Au^III^X_2_(MSDT) (Figure 2C) compounds have been tested on myelogenous leukemia cell lines such as K562 cells, and the results showed that Au^III^X_2_(MSDT) inhibits cell growth in all tested myeloid cell lines with IC_50_ values ten-fold lower than those of the palladium(II) analogs (Nobili et al., 2010). Au^III^Cl_2_- dimethoxydiphenyltrichloroethane (DMDT) (Figure 2B) exhibits the best *in vitro* cytotoxic activity towards the androgen-resistant prostate cancer PC3, by inducing apoptosis, and APO2.7 (a mitochondrial membrane protein exposed on the surface of cells undergoing apoptosis) expression (Cattaruzza et al., 2011). Au^III^Br_2_- DMDT (Figure 2D) has been shown to inhibit cell proliferation in several breast cancer cell lines. It exhibits greater *in vitro* cytotoxic activity than cisplatin (Milacic et al., 2006). Au^III^Br_2_-ethylsarcosinedithiocarbamate (ESDT) (Figure 2E) shows high toxicity but causes no significant changes in both urinary and renal cortical biomarkers, accounting for the almost complete lack of nephrotoxic side effects (Ronconi et al., 2006). Gold compounds, such as Na_3_Au(S_2_O_3_)_2_·2H_2_O, induce the release of IL-8 from monocyte-derived dendritic cells (MoDCs), PBMCs, or THP-1 cells. Gold compounds result in modest dendritic cell maturation via increased membrane expression of CD40 and CD80 (Rachmawati et al., 2015). Additionally, other gold compounds such as [Au(en)_2_]Cl_3_ (Figure 2F) play a role in inducing anticancer activity (Isab et al., 2011; Garcia-Moreno et al., 2015). Accordingly, several studies outlined in the following sections implicate a complex interplay between novel anticancer metal complexes, including gold compounds, and the anticancer immune response, both of which directly affect cancer cell immune responses (Terenzi et al., 2016). At present, Au(I) and Au(III) compounds are promising candidates for anticancer therapy (Table 1).

**TABLE 1:** Experiments involving gold compounds for cancer therapy.

Gold compounds	Mechanism	Cancer types	Subjects	References
Au^III^Cl_2_(DMDT)	Alters mitochondrial functions, stimulates ROS, and strongly inhibits the activity of the selenoenzyme TrxR	Prostate cancer	PC3 cells	Cattaruzza et al. (2011)
Au^III^(MSDT)	Promotes tumor cell apoptosis	Cervical carcinoma, Leukemia	HeLa cells, HL60 cells	Giovagnini et al. (2005)
Au^III^X_2_(MSDT)	Promotes early apoptosis, induces cell cycle perturbations, and high DNA fragmentation	Leukemia	K562 cell	Nobili et al. (2010)
Au^III^Br_2_(DMDT)	Inhibits proteasome activity, accumulates protein p27, and induces massive apoptosis	Breast cancer	Nude mice	Milacic et al. (2006)
Au^III^Br_2_(ESDT)	Induces a dramatic inhibition of DNA synthesis; lack of nephrotoxic side-effects	Breast cancer	Mice	Ronconi et al. (2006)
Na_3_Au(S_2_O_3_)_2_·2H_2_O	Release IL-8 from MoDCs, PBMCs, or THP-1 cells	Leukemia	THP-1 cells	Rachmawati et al. (2015)
[Au(Spyrimidine)(PTA-CH2Ph)]Br	Inhibits colon cancer cell proliferation	Colon cancer	Caco-2/PD7 cells	Garcia-Moreno et al. (2015)
[Au(en)2]Cl3	Induces cell cycle blockage, interrupts the mitotic cell cycle, apoptosis, or necrosis	Prostate cancer, Gastric carcinoma	PC-3 cells, SGC-7901 cells	Isab et al. (2011)
Au(C-C-2-NC_5_H_4_)(PTA)	Produces ROS, which triggers necroptosis	Colorectal cancer	Caco-2 cells	Marmol et al. (2017)
	Induces necroptosis dependent of TNF-α and TNFR1 binding, activates RIP1 and NF-κB signaling			

X = Cl, Br; PTA = 1,3,5-triaza-7-phosphadamantane.

### Gold Compounds and Innate Immunity

Innate immunity forms the first line of defense in the human immune system. NK cells are natural immune effector cells with a direct killing function that plays a key role in the clearance of tumor cells. Metal drugs can upregulate signals on cancer cells that are perceptible to the NK cell compartment, such as the NKp30 ligand B7-H6F (Cao et al., 2015). NKG2 families, such as NKG2D, may also be highly expressed by several T cell subsets, including NKT and γδ T cells, and activated CTLs, which are directly cytotoxic to cancer cells (Jelencic et al., 2017). Metal drugs damage DNA directly or indirectly by generating redox products, such as ROS (Lai et al., 2015) and NKG2D, and by activating DDR. These phenomena effectively render gold compound-treated cancer cells generally more responsive to both innate and adaptive immunity in an MHC-independent manner. Gold compounds such as Au(C-C-2-NC_5_H_4_)(PTA) (Figure 2G) induce colorectal carcinoma cell death via ROS-mediated necroptosis by activating TNF-α and NF-κB signaling (Marmol et al., 2017). Gold(I) compounds may exert an immunosuppressive role by inhibiting IB kinase activation and promoting cell apoptosis (Jeon et al., 2003; Kim et al., 2004). Au(I) compounds reduce TNF-α via the action of certain immune cells, including neutrophils and macrophages, and Au(I) compounds enhance leukocyte adhesion to endothelial cells, both of which are important in the pathogenesis of rheumatoid arthritis (RA) (Madeira et al., 2012). Au(I) can oxidize inside phagocyte lysosomal compartments, resulting in Au(III), which plays the role of a major hapten that acts synergistically in innate immunity (Baeck and Goossens, 2012). An experiment using the p38 MAPK blocker SB203580 has shown that it strongly suppresses the gold-induced IL-8 production by THP-1 cells, indicating that innate signaling by gold involves p38 MAPK phosphorylation. The innate triggering capacity of the metal may contribute to its irritant properties and also play a role in the induction of autoimmunity (Mutter, 2011). Elie et al. investigated the anti-metastatic effects of gold compounds in renal cancer cells and revealed strong inhibition of several cytokines (IL17A, IL-8, IL-6, and IL-5) by gold compounds (Elie et al., 2018). Gold compounds, such as Na_3_Au(S_2_O_3_)_2_·2H_2_O, induce the release of IL-8 from MoDCs, PBMCs, or THP-1 cells. Furthermore, they have been shown to result in modest dendritic cell maturation via increased membrane expression of CD40 and CD80. Various studies have shown that gold compounds can elicit an innate immune response, which can be ascribed to the triggering of TLR3 rather than TLR4 (Rachmawati et al., 2015). Additionally, gold nanoparticles (AuNPs) efficiently deliver synthetic thiolated CpG oligodeoxynucleotides (ODNs) into cultured cells and increase the expression of proinflammatory cytokines (Chen et al., 2014).

### Gold Compounds and Adaptive Immunity

Adaptive immunity against an infection targets only a specific pathogen. It usually develops after stimulation with microorganisms and other antigenic substances and reacts specifically with antigens. Gold compounds are known to induce innate immune responses; however, these responses may contribute to adaptive immune responses, as reflected in cases of skin and mucosal allergies (Rachmawati et al., 2015). Gold compounds contribute to the frequent development of adaptive immunity by directly triggering TLR3 and increasing the expression of downstream mediators (Martin et al., 2011). Ma et al. attempted to mimic the function of an adaptive immune response built from AuNPs. These mechanisms mimic the differentiation and coordinated interaction of three key immune cells, namely the T lymphocytes, B lymphocytes, and dendritic cells, upon exposure and subsequent response to an invading pathogen. The results show that adaptive immunity can sense and clear the corresponding pathogen and simultaneously respond more effectively and rapidly upon invasion by the same pathogen for a second time (Ma et al., 2020).

### Gold Compounds Support Cancer Cell Antigenicity

**FIGURE 3:**
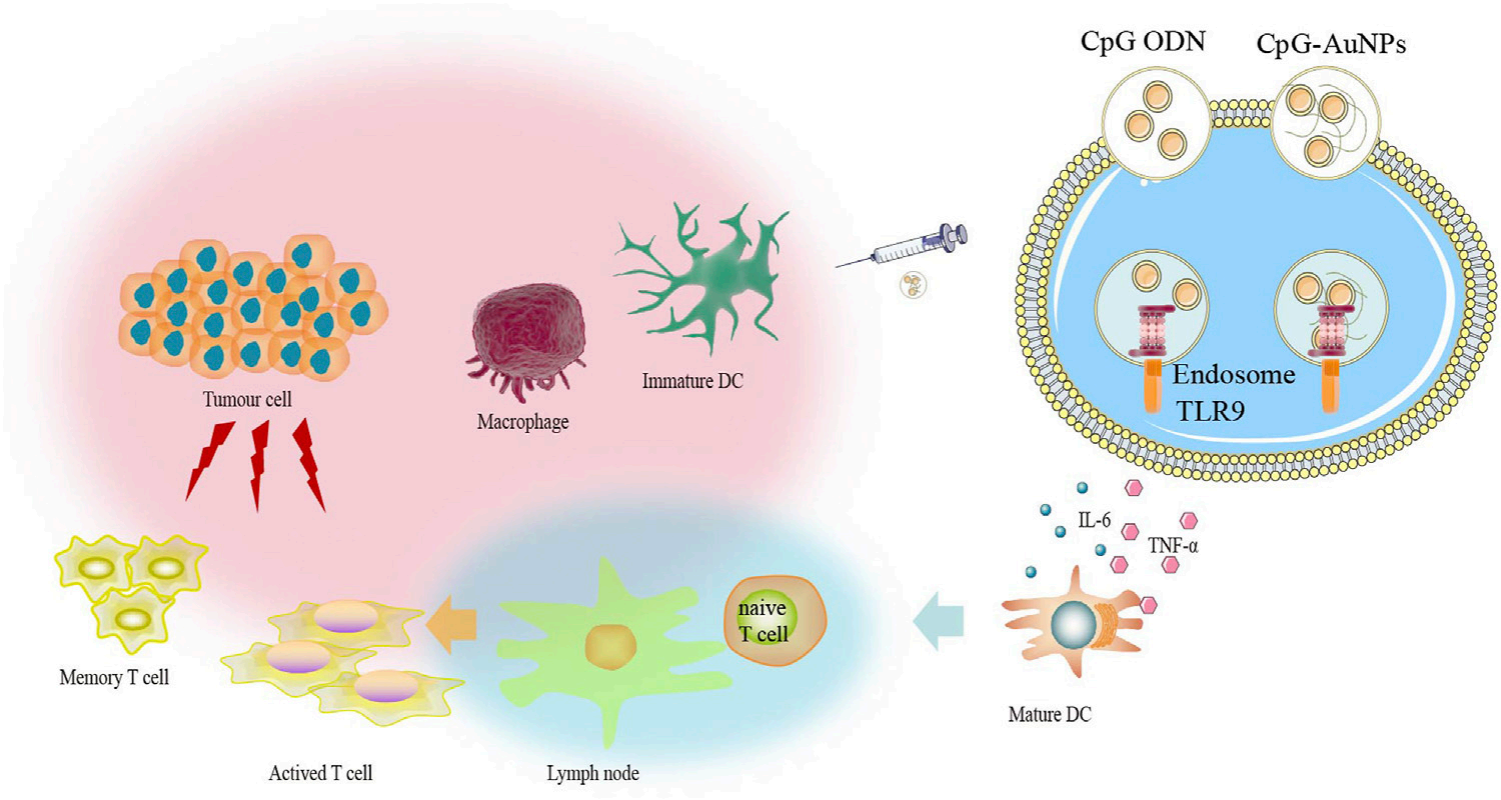
The CpG delivery system based on gold nanoparticles (AuNPs) can induce strong immune response even at low dose of CpG via TLR9 interaction.

Since the discovery of the first tumor antigen in 1991 (van der Bruggen et al., 1991), more than 100 tumor antigens have been identified successively (Cheever et al., 2009). Tumor antigens are only expressed in tumor tissues but not in normal tissues and include antigens produced by the integration of oncogenic viruses into the genome and mutant proteins. New antigens not only have high specificity, but also have strong immunogenicity because they have not been screened during negative selection in the thymus (Rizvi et al., 2015). Since virus-mediated tumors account for only a small proportion of all tumor types, new antigens derived from mutations are the most ideal targets for immunotherapy (McGranahan et al., 2016). Currently, AuNPs used in vaccination are being combined with other immunostimulants, especially cytosine guanine dinucleotide (CpG). The suitability of CpG is being assessed for use in human vaccines as adjuvants as they are strong immunogenic DNA fragments that distinguish friend or foe recognition systems between mammalian DNA and bacteria (Staines, 2005). Gold compounds could be leveraged to facilitate delivery of the ovalbumin peptide antigen (OVA) as well as the CpG adjuvant and enhance their therapeutic effects in tumor models. Gold NP delivery of OVA (AuNP-OVA) and CpG (AuNP-CpG) increases the efficacy of both agents and induces strong antigen-specific responses. Furthermore, AuNP-OVA delivery alone is adequate for producing antigen-specific responses, leading to anticancer activity and prolonged survival (Almeida et al., 2015). Another method of activating immunocompetent cells with gold is to conjugate AuNPs with CpG oligodeoxynucleotides (ODNs) (Wei et al., 2012). Several studies have shown that AuNPs possess a strong potential to activate cellular immunity and immunological memory to promote immune response. Gold conjugated to CpG ODNs can enhance intracellular penetration and increase the secretion of proinflammatory cytokines, such as TNF-α and IL-6. Particularly, AuNPs coupled with CpG ODNs are much more immuno-stimulatory than native CpG ODNs. AuNPs combined with CpG show enhanced efficiency in cellular delivery and the immuno-stimulatory effect of CpG because of the high cellular uptake of CpG-AuNPs. Furthermore, CpG-AuNPs could potentially be used for immunotherapy *in vivo*. Another study revealed that TNF-α levels stimulated by CpG-AuNPs was approximately 15-fold higher than that stimulated by CpG ODNs, indicating that high cellular uptake of CpG-AuNPs was significantly associated with immunostimulatory activity. Moreover, AuNPs had nearly no effect on TNF-α secretion, indicating that CpG ODNs may elicit immunostimulatory activities (Luo et al., 2019). Further research is essential for better understanding the mechanism underlying the combined use of AuNPs and CpG ODNs (Wang et al., 2016). The CpG delivery system based on AuNPs can induce a strong immune response even at a low dose of CpG by effusively interacting with TLR9 (Figure 3). Immune cells, including Th cells, CTLs, and macrophage polarization, can be affected by cytokine profiles, thereby maintaining a balance between drug resistance and immune-stimulatory behaviors. Gold compounds, such as RANCE-1, warrant further investigation to enhance the antigenicity of cancer cells (Madeira et al., 2012). T cells are currently considered the only cells that can specifically kill tumor cells. DCs present antigens to T cells, which induces the activation and proliferation of T cells, including CD4^+^ helper T cells and CD8^+^ killer T cells. Tumor antigen-specific T-cells are important players that facilitate tumor treatment. Currently, the ongoing anti-tumor immunotherapy employing T cells can be divided into two categories: T-cell adoptive therapy, which mainly includes Professor Steve Rosenberg’s tumor-specific T cell expansion from patients' tumor-infiltrating lymphocytes (TILs) for reinfusion, and ectopic expression of receptors that can recognize tumor antigens on the surface of normal T cells by gene modification, including T cell receptor (TCR) - modified T cells (TCR-T) and chimeric antigen receptor (CAR) - modified T cells (CAR-T). The immune system identifies cancer cells based on the detection of “self” antigens with deregulated expression including differentiation and germ cell antigens, all of which belong to the class of “tumor-associated antigens” (TAA) (Lewis et al., 2003). The expression of these tumor-associated antigens in tumor cells is stimulated by metal drugs that enhance their antigenicity. MHC class I presentation of viral peptides or mutation/fusion protein-derived immunogenic neoantigens by malignant cells determines the response time of T cells and the tumor rejection response (Braunlein and Krackhardt, 2017). This is similar to anticancer metal drugs that are considered mutagenic. Metal drugs induce chromosomal aberrations, especially during the development of resistance (Castedo et al., 2006). As a cell that can specifically kill tumor cells, adoptive transfusion of T cells has created a precedent for tumor immunotherapy. As early as 1985, Rosenberg et al. found that injection of IL-2 and LAK cells *in vivo* could cause metastatic melanomas to regress for a long time, suggesting that there was specific T cell expansion (Rosenberg et al., 1985). To produce specific T cells for other tumors, a corresponding technology was developed. First, the researchers inserted the normal TCR, which can recognize tumors and present tumor antigens through MHC molecules, into the T cells of patients, which were subsequently expanded and reinfused into the patients. Furthermore, it was revealed that recognition of gold by T cells consisted of MHC-restricted and MHC-independent pathways (Hashizume et al., 2008). Researchers have also shown that T cells specifically expressing TCR that recognize MART-1 melanoma can induce tumor regression for the first time in 2006 (Morgan et al., 2006).

Known tumor antigens can be produced by synthesizing peptides or nucleic acids encoding specific tumor antigens (Anguille et al., 2012; van den Ancker et al., 2010). For undefined tumor antigens, DC vaccines are usually prepared by repeatedly freezing and thawing tumor cells, using apoptotic tumor cells or apoptotic bodies to obtain tumor antigens, or fusing tumor cells with DCs (Yao et al., 2014). Tumor exosomes play an important role in determining organ metastasis preference and early diagnosis because they contain extensive information about tumor cells (Hoshino et al., 2015; Zhang et al., 2015) and can also be used as a source of tumor antigens to load DCs (Romagnoli et al., 2014). The advantage of DC loading with different forms of tumor cell-derived antigens is that they can obtain all the antigens of tumor cells; however, this may also cause the presentation of unrelated antigens or autoantigens, induction of tolerance, or autoimmunity (Chiang et al., 2015). However, necrotic tumor cells can release a large number of HSPs and HMGB1, which promote the maturation of DCs. Apoptotic tumor cells can express a large amount of calreticulin (CRT) on the surface of DCs and release HSPs and HMGB1 (Guo et al., 2014; Chiang et al., 2015), which can effectively fight against IL-10 or TGF-β, which have a negative regulatory effect. Therefore, studies have shown that DCs loaded with necrotic or apoptotic tumor cells can induce an effective CTL response (Ruben et al., 2014). To induce DCs to uptake tumor antigen *in vivo*, antibodies against DC receptor can be used to form chimeric protein with a specific antigen. Some studies have found that the antibody cross-linked antigen of endocytosis receptor on the surface of DCs can increase the efficiency of DCs to activate T cells by 100 times more than that of liquid antigen, thus effectively promoting the anti-tumor effect of DCs (Mahnke et al., 2000). However, it should be noted that different DC subpopulations can stimulate different immune responses, so CD205 antibody recognizing CD8^+^ DCs and 33D1 antibody recognizing CD8^−^ DCs can ultimately stimulate different anti-tumor immune responses. CD8^+^ DCs can present antigens through MHC class I and MHC class II molecules, while CD8^−^ DCs can only present antigens through MHC class II molecules (Soares et al., 2007). At the same time, targeting DCs is not limited to antigen delivery. Researchers have also detected the effects of different costimulatory signals on DCs, such as DECTIN-1, DC-SIGN, and CD40, which also promote the activation of DCs. Gold compounds selectively kill cancer cells based on enhanced ROS production, but at the same time protect phagocytic cells such as DCs in the TME (Oommen et al., 2016). Na_2_Au(I)TM hinders T cell receptor-mediated antigen recognition by mouse CD4^+^ T cell hybridomas specific for antigenic peptides. However, it should be noted that different activation signals can cause polarization of the DC function. For example, DC-ASGPR can cause DCs to secrete IL-10 and negatively regulate the anti-tumor effects of DCs (Kochenderfer et al., 2012). Additionally, DNA damage of cancer cells by anticancer drugs, such as metal compounds, induces cell-autonomous production of pro-inflammatory cytokines (Wan et al., 2012; Beyranvand Nejad et al., 2016).

### Gold Compounds Enhance the Anti-tumor Immune Response by Enhancing Immunogenicity

In healthy adults, cells are lost every second due to programmed cell death, even in the absence of disease or systemic inflammatory responses (Kroemer et al., 2013). This phenomenon is a self-balancing cell death, usually through apoptosis, which is considered to be immune tolerant (Green et al., 2009; Galluzzi et al., 2012). Immunogenic cell death is a form of cell death that can stimulate the immune response to anti-death cell antigens, especially those derived from tumor cells (Zitvogel et al., 2011). Clinical evidence shows that a tumor-specific immune response can serve as a determinant for the application of traditional cytotoxic drugs in anticancer treatment. Gold compounds in combination with CRISPR/Cas9-mediated disruption of PD-L1 and mild hyperthermia induce the activation of immunogenic cell death (Tang et al., 2021). Additionally, gold compounds eliminate primary tumors and induce immunogenic cell death via the release of damage-associated molecular patterns (DAMPs), activation of effector cells, and induction of dendritic cell maturation. These phenomena, in a coordinated manner, eventually evoke systematic anticancer immune responses (Liang et al., 2018).

#### Induction of Calreticulin Exposure on the Surface of Dying Cells

CRT is a cytoplasmic calcium-binding protein that is the most abundant protein in the endoplasmic reticulum (Biwer and Isakson, 2017). It is known to play a pivotal role in increasing the immunogenicity of cancer. Anticancer drugs, including gold compounds, not only induce the transfer of CRT from the cytoplasm to the cell membrane of dying cells, but also target CRT to interfere with cancer cell protein folding. The death of immunogenic cells results in the induction of CRT exposure, and the activation of the immune response is closely related to the expression of CRT on the surface of tumor cells. During immunogenic cell death, CRT exposure occurs at a relatively early stage, when the cells still have normal morphology and lack phosphatidylserine exposure. Therefore, one of the potential ways to achieve anti-tumor therapy is to increase the exposure of CRT on the cell surface. CRT is a key factor that determines the immunogenicity of dead cells. The uptake and phagocytosis of apoptotic cells and cancer cells involves the assistance of CRT on the cell surface. Moreover, the body’s anti-tumor immune response can be stimulated by CRT purified from tumors. The possible underlying mechanism might involve binding of the tumor antigen polypeptide to CRT, which results in cell membrane resurfacing and presentation of the antigen peptide to the antigen-presenting cells during CRT eversion to the cell membrane. This ultimately stimulates the body’s anti-tumor immune response (Gardai et al., 2005) when CRT is recognized and bound by cells with membrane penetrating receptor CD91 and transmits phagocytic signals (Kim TG. et al., 2010), enhancing the immune recognition and clearance of tumor cells by DCs. Furthermore, gold compound nanoclusters (AuNCs) specifically recognize surface-bound CRT and serve as fluorescent bio-probes (Ramesh et al., 2016).

#### Induction of ATP Secretion by Dying Cells

ATP is the most abundant intracellular metabolite and an important autocrine/paracrine messenger. It transmits signals by binding to purine receptors of ion transporters (P2X) or metabolizers (P2Y) (Zhao et al., 2020). Additionally, many chemotherapeutic drugs promote apoptotic cells to release ATP, which then binds to P2X7 on the surface of immune cells [(Zitvogel et al., 2012)]. When ATP binds to P2RX7 on DCs, K^+^ and Ca^+^ efflux further activates the NLRP3 inflammasome, stimulating proteolysis maturation and secretion of IL-1β and IL-18, which ultimately enhances the body’s anti-tumor immune response (Andersson and Tracey, 2011).

#### Induction of High Mobility Group Box 1 Release From Dying Cells

High mobility group box 1 (HMGB1) is usually expressed in nucleated cells and is the most abundant non-histone nuclear protein. It is actively secreted by innate immune cells in response to pathogens and released when cells are in the initial or secondary necrosis (Sharabi and Ghera, 2010); moreover, it exhibits nuclear retention by inhibiting its release from activated macrophages (Zetterstrom et al., 2008). HMGB1 can bind to Toll-like receptor 4 (TLR4), promote the release of proinflammatory cytokines from monocytes/macrophages and regulate the function of endothelial and cancer cells (Ladoire et al., 2011). In a co-culture experiment employing both TLR4 and HMGB1, DCs halted the cross presentation of tumor antigens (Apetoh et al., 2007). Furthermore, *in vitro* experiments involving the application of HMGB1 to DCs expressing TLR4 showed an increased production of IL-1β, resulting in abrogation of lysosomal digestion of phagocytic tumor antigen, which is a prerequisite for cross presentation. Thus, DCs rely on the combination of TLR4 and HMGB1 to facilitate their function of antigen presentation.

#### Inducing Expression of Heat Shock Proteins in Apoptotic Tumor Cell

Heat shock proteins (HSPs) can form peptide HSP complexes with tumor antigen peptides, which can improve the uptake of tumor cells and antigen presentation by DCs (Miragem and Homem de Bittencourt, 2017; Bryant et al., 2019). Chemotherapy drugs induce apoptosis of gastric cancer cells exposed to HSP70, accompanied by increased phagocytosis and antigen presentation of apoptotic cells, increased secretion of IL-12, and activation of heat shock protein (HSP) promoter-driven protein expression (Nakatsuji et al., 2017). Apoptotic myeloma cells treated with the proteasome inhibitor bortezomib have been shown to transmit activation signals to DCs, which depends on the direct contact between DCs and dead tumor cells and the exposure of HSP90 on the surface of dead cells (Bryant et al., 2019).

#### Induction of NKG2D Expression on Tumor Cell Surface

**FIGURE 4:**
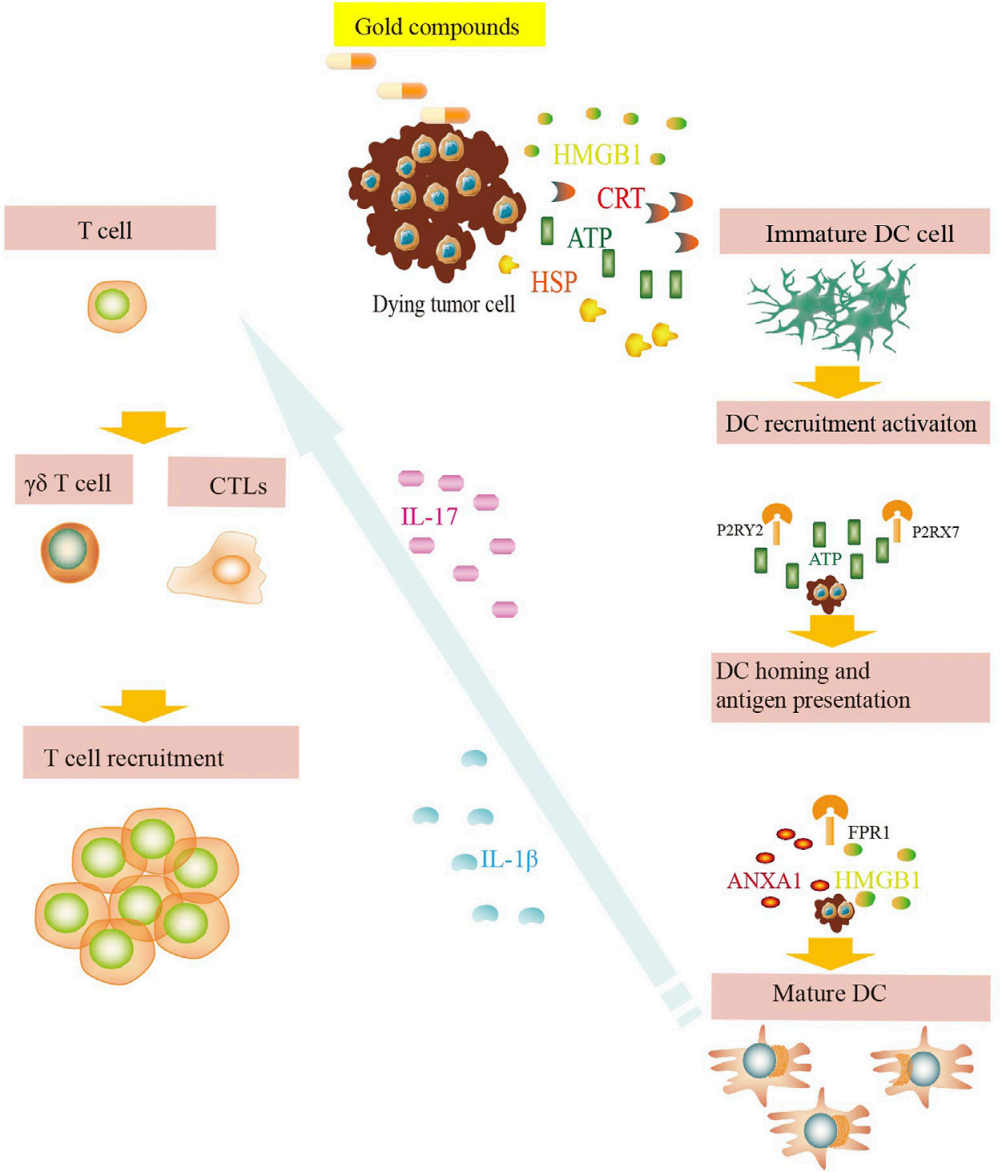
DAMP signals during exposure to gold compounds. Dying tumor cells expose CRT on their surface and release ATP, HMGB1, HSPs, etc. and bind to their respective surface receptors on myeloid or lymphoid cells. These danger signals favor the uptake of debris and dead cells by antigen presenting cells, including DCs. The dying tumor cells induce release of IL-17 and IL-1β, which favor the recruitment of T cells. These phenomena eventually lead to the priming of an anticancer immune response.

NK group 2 member (NKG2D) is expressed in NK cells, CD8^+^ T cells, and γδ T cell active receptors, which recognize the corresponding ligands, such as major histocompatibility complex class I chain-related molecule A or B (MICA/B) and UL16-binding protein (ULBP) (Perez et al., 2019; Cadoux et al., 2021). Tumor cells can escape NKG2D- mediated immune monitoring by downregulating the expression of NKG2D ligands on the cell surface. Some chemotherapeutic drugs can upregulate the expression of NKG2D ligands in tumor cells, thus enhancing the recognition and killing effect of effector lymphocytes, such as NK cells, on tumor cells (Butler et al., 2009). The activation of ataxia telangiectasia-mutated (ATM)/ataxia telangiectasia-Rad3-related (ATR) signaling pathway may be one of the mechanisms of DNA damage induced by chemotherapeutic drugs (Sayitoglu et al., 2020). Gold compounds effectively guide NK cells to concentrate around cancer cells for effective gene therapy without affecting cell activity (Zhuo et al., 2019) (Figure 4).

### Effects of Anticancer Metal Drugs on Immune Cells

#### Suppressor Regulatory T Cells

Regulatory T cells (Tregs) are a subset of CD4^+^ T cells with immunosuppressive functions. Its phenotype is CD4^+^, CD25^+^, Foxp3^+^, which can inhibit the activities of CD4^+^ and CD8^+^ T cells as well as the DC and NK cells. Au(I) compounds show high activity in inhibiting cancer cell activation and proliferation (Kiely et al., 2000). Studies have shown that there is enhanced aggregation of Treg population and inhibition of anti-tumor immune response in patients with a variety of solid tumors and malignant hematomas (Wada et al., 2009). Furthermore, low-dose chemotherapy drugs can inhibit the function of Tregs *in vivo*, reduce their population, and increase the number of antigen-specific T-cells. However, they have few side effects on other immune cells *in vivo*, thus enhancing the body’s immune response to tumors (Vicari et al., 2009; Ishizaki et al., 2011).

#### Inhibition of Myeloid Derived Suppressor Cells

Myeloid-derived suppressor cells (MDSCs) are a group of immature myeloid cells that can inhibit the activity of T cells. MDSCs accumulate in the tumor host and inhibit anti-tumor immune response. Low-dose chemotherapeutics are generally selective for inhibiting MDSCs, which can significantly reduce the number of MDSCs in tumor-bearing mice and enhance tumor-specific CD8^+^ T cell responses without any significant decrease in other immune cells (Ko et al., 2007; Ishizaki et al., 2011). However, the number of MDSCs in the body increases when high-dose chemotherapy drugs are used (Kim HS. et al., 2010). Therefore, it can be concluded that the intrinsic characteristics and dose of chemotherapy drugs might be responsible for determining their impact on MDSCs.

#### Enhancement of the Function and Number of DCs

Rachmawati et al. analyzed the effect of gold compounds on innate immune cells and showed that the gold compound Na_3_Au(S_2_O_3_)_2_·2H_2_O can induce moderate DC maturation via TLR3 signaling (Rachmawati et al., 2015). Furthermore, some low-dose chemotherapy drugs can enhance the function of DCs by upregulating the expression of important functional molecules, including antigen processing machinery (APM), MHCII, and costimulatory molecules, including CD40, B7-1, B7-2, and IL12 (Shurin et al., 2009). Certain chemotherapeutic drugs have also been shown to induce DC maturation without causing significant cell death. Most topoisomerase inhibitors and anti-microtubule drugs have been shown to exert this effect (Le Naour et al., 2021). The number of immature DCs in peripheral blood has also been shown to increase significantly after the use of a large-dose quantitative therapy. TLR3 agonists induce DC maturation and migrate to the lymph nodes. These DCs then amplify antigen-specific CD8^+^ T cells. Therefore, it is safe to conclude that the effect of chemotherapy drugs on DC proliferation depends on the degree of lymphocyte inhibition. The greater the drug dose, the stronger the inhibition of lymphocytes and more significant the effect on DC proliferation (Rinaldi et al., 2021).

### Nanoparticles of Gold Compounds and the Immune Response

**FIGURE 5:**
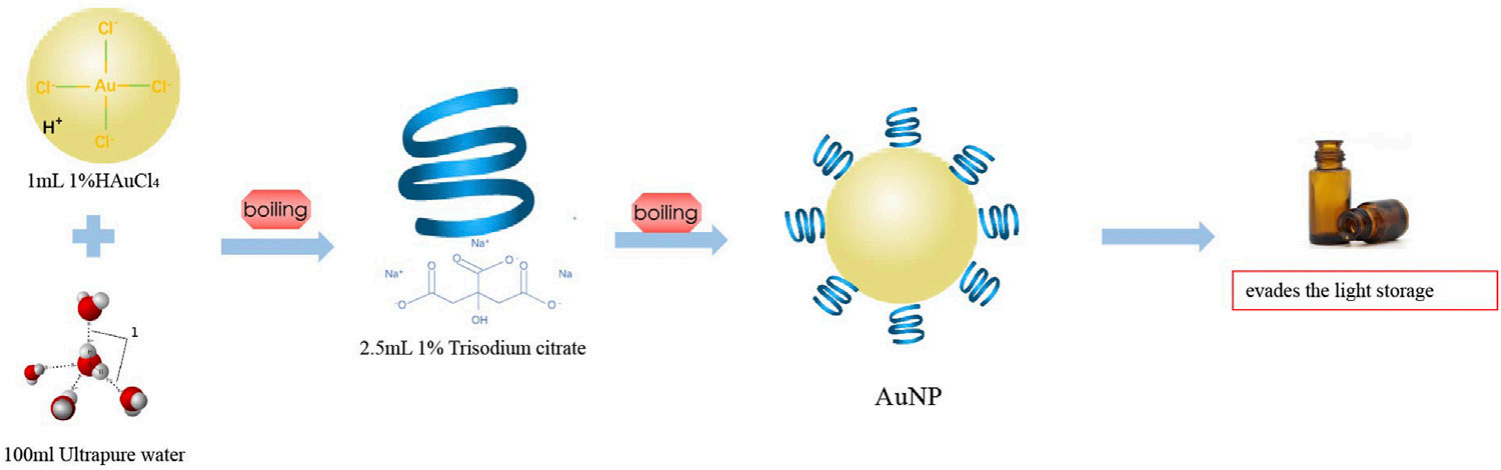
Schematic representation of gold nanoparticle (AuNP) production.

Among the many nanomaterials being developed for nanomedicine applications, AuNPs have been promising in the treatment of cancer owing to their unique properties. AuNPs are small gold particles with diameters in the range of 1–100 nm. AuNPs have good stability because of their small size and surface, optical effects, and their unique biological affinity. The process of AuNP production was as follows: 1 ml 1% HAuCl4 solution was added into 100 ml ultra pure water heated until boiling, and 2.5 ml 1% trisodium citrate solution was quickly added under strong stirring. The solution was boiled until it turned wine red. The solution was nano gold reserve sol (concentration was 56.02 mg/L) and stored in dark at 4°C (Figure 5). AuNPs are the most thoroughly investigated metal-based NPs for cancer therapy. The clinical effectiveness of metal anticancer drugs is limited due to certain factors; hence, NP-based platforms have been employed to encapsulate anticancer drugs and selectively deliver them into tumors to overcome poor tumor specificity (Vila and Walcarius, 2020). NPs concerning metal nanomaterials consist of a metal core (e.g., AuNPs) and are known to interact with both the innate and adaptive immune systems, which can lead to hypersensitivity, immunogenicity, and autoimmunity. In recent years, there has been an increasing interest in NPs with increasing evidence supporting their anticancer effects (Shi et al., 2017), and the effects of AuNPs on the activation of the immune system (Dykman and Khlebtsov, 2017). Metal-based NPs, including AuNPs, provide a good foundation for the development of functional cancer immunomodulators. Nanotechnology has the following advantages in cancer immunotherapy: 1) drugs can be delivered to immune cells and tissues that are easily targeted by nanoparticles; 2) the interaction between nanoparticles and immune cells or organs can be regulated by modifying drug nanoparticles; 3) nano drug carriers can improve the pharmacological properties of drugs and prevent the premature release and degradation of drugs; 4) nanoparticles can be designed as drug carriers with specific responses to achieve targeted drug delivery and reduce off-target toxicity; and 5) targeted drug delivery of nanoparticles, combined with controlled and local drug release, can achieve economical dosage of immune checkpoint inhibitors or activate immunotherapy only at the expected site of action, thus reducing the non-specificity-associated safety risks with immunotherapy. Cancer treatment involves not only metal-based drugs, but also inorganic nanoparticles that have been tailored as therapeutic or imaging agents, including nanosemiconductors, carbon nanotubes, and those derived from metals. The combination of organometallic drugs and inorganic metals may promote the clinical transformation of related metal drugs, including gold compounds.

#### AuNPs as Inducers for Photothermal Therapy

PTT relies on materials with high photothermal conversion efficiency and is a promising anti-tumor therapy technique. It has been proven that PTT can induce immunogenic cell death (ICD) in cancer cells, and tumor-associated antigens released during PTT can stimulate phagocytosis of antigen-presenting cells. AuNPs are widely used as PTT agents. Ma et al. reported a PTT strategy based on near-infrared II that induces cancer cells to produce more uniform and stronger ICD, triggering an immune response to prevent tumors (Ma et al., 2019). Nam et al. developed novel polydopamine-coated AuNPs as photothermal reagents with high photothermal efficiency, which can induce cancer cells to produce ICD *in situ*. Combined with traditional chemotherapy drugs, PTT can further enhance the anti-tumor immune effect in the whole body and inhibit the development of primary tumors and metastasis (Rinaldi et al., 2021). Zhang et al. developed a nanoparticle platform for PTT-induced tumor immunotherapy. It involves the incubation of AuNPs with cancer and DC cells to increase their immunogenicity (Zhang et al., 2019).

#### Gold Nanoparticles as Antigens and Immune Adjuvants to Regulate Dendritic Cells

**FIGURE 6:**
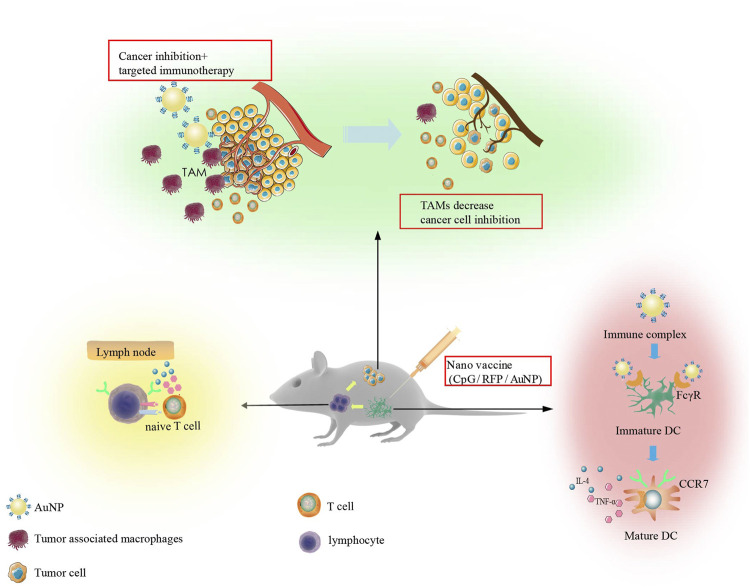
Immunotherapy strategy designed on the basis of *in vitro* studies. The synthesized Nano vaccine can regulate dendritic cells, enhance their migration to lymph nodes, and result in T cell activation. This ultimately reduces M2 tumor associated macrophages producing efficient anti-tumor immunity.

Immune adjuvants are non-specific immune enhancers that can be injected together with or before the antigen to enhance or modulate the type of immune response. Cytosine-phosphate-guanine oligonucleotide (CpG ODN) is an efficient immune adjuvant that combines with TLR9 on APCs to enhance specific and non-specific immune responses. AuNPs can be used as antigens and immune adjuvants. In tumor therapy, the co-delivery of antigen and adjuvant as a nano-vaccine has been proven to have a good synergistic effect. The presence of antigens and adjuvants in APCs can result in stronger and longer immune activity. Lee et al. used AuNPs to transfer antigens and adjuvants together as a nano-vaccine (CpG/RFP/AuNP) to localize local lymph nodes. With the help of computed tomography (CT) imaging features of AuNPs, the characteristics of metastasis to local lymph nodes and long-term conditions were observed. Cross-presentation of dendritic cells and further cooperation of immune cells can stimulate a strong immune response and effectively inhibit tumor growth. Nano-vaccines have also been demonstrated to curtail lung metastasis by the Th1 helper-facilitated T cell immune response (Lee et al., 2012). Another study involving the synthesis of AuNPs (OVA-AuNCs-CpG) loaded with antigen OVA and adjuvant CpG showed that AuNPs promoted the uptake of OVA and CpG by APCs (Figure 6).

### Gold Nanoparticles as Regulators of Macrophages

Tumor-associated macrophages (TAMs) have positive and negative regulatory effects on cancer cells. M1 macrophages can resist the invasion of pathogens and kill cancer cells, while M2 macrophages can promote the growth, invasion, and metastasis of cancer cells. Knocking down M2 macrophage-associated protein expression or direct removal of M2 macrophages can effectively improve the immunosuppressive microenvironment. Furthermore, AuNPs loaded with EGFR siRNA to silence the expression of EGFR in M2 macrophages and lung cancer cells inhibited angiogenesis and produced lasting anti-tumor immune effects (Conde et al., 2015) (Figure 6).

The use of AuNPs for anticancer treatment has attracted the attention of many researchers. Cruz et al. demonstrated the synthesis of 13 nm AuNPs conjugated to prostate cancer-associated antigen peptides for tumor immunotherapy (Cruz et al., 2011). These antigen-conjugated AuNPs were shown to be internalized by DCs, resulting in the production of an obvious immune response that was not produced in case of the native antigen alone. Saha et al. found that in malignant progression, unmodified 20 nm AuNPs could disrupt the crosstalk between pancreatic cancer-associated fibroblasts and pancreatic cancer cells to alter the TME in pancreatic ductal adenocarcinoma (PDAC) (Saha et al., 2016). ER stress, identified as a probable mechanism hindering cancer cells, disrupts the directional communication between pancreatic cancer cells (PCCs) and pancreatic stellate cells (PSCs) by altering the cell secretome. Cytokines such as IL-8 and GM-CSF are important immunomodulators that are downregulated by AuNPs, suggesting a potentially improved outcome of immune therapy (Saha et al., 2016). Triple-negative breast cancer research has demonstrated the theragnostic capability of actively targeted, site-specific multi-branched gold nanoantennas *in vitro* (Webb et al., 2017). AuNPs can efficiently deliver synthetic thiolated CpG ODNs into cultured cells and increase the expression of proinflammatory cytokines, including TNF-α, IL-6, IL-12, and MCP-1 (Chen et al., 2014). Webb et al. developed AuNPs by combining diagnostic and therapeutic properties of an antibody targeting PD-L1, which is widely used as a therapeutic tool for cancer and other diseases (Webb et al., 2017). Furthermore, the same NP were administered to ICR mice. In a recent paper, Lee et al. described promising radionuclide-embedded GNP that provoked DC maturation and anti-tumor immunity to levels comparable to or even higher than those of DC pulsed with tumor lysates (Lee et al., 2018), implicating the potential for therapeutic application of these nanomaterials. Therefore, AuNPs can be used to improve the effect of immunotherapy and reduce the toxic and side effects of the treatment, thereby overcoming the shortcomings of traditional cancer treatment. These strategies of employing AuNPs provide novel ideas for promoting clinical cancer immunotherapy and provide important directives for the development of new personalized cancer treatment modalities.

## Conclusion

The immune system plays a key role in the adverse effects of the gold compounds. First, several adverse effects can be related to immunosuppression, such as impairment of macrophages and T and B cells. Second, immunestimulating reactions can also occur. The most frequent immune-stimulating reactions of gold compounds involve diverse types of skin reactions. Contact dermatitis by gold compounds is probably caused by slow ionization of gold upon contact with the skin and subsequent absorption and haptenization as well as modification of otherwise non-immunogenic cellular structures by antigenic compounds including metal ions, leading to an immune response.

There is a growing recognition that the cytotoxic effect of immune cells might cause transient immune depletion, which might provide the opportunity to protect against cancer immune evasion and induce a phase of renewed anti-tumor immune response. The effects of gold-based cancer therapy on the immunological aspects of cancer have been extensively studied, with gold compounds emerging as potent anticancer agents. Gold compounds have been shown to enhance the anti-tumor immune response and affect the population of immune cells. Moreover, nanoparticles of gold compounds can be used to improve the efficacy of immunotherapy and reduce associated toxicity and side effects. The combination of gold compounds with the immune system has demonstrated promising results in both *in vivo* and *in vitro* studies. Furthermore, based on preclinical data, these effects provide an ideal opportunity for exploring the combination of anticancer gold compounds and immunotherapeutic interventions. These findings imply the emergence of a new area for gold compounds that potentially serve as novel anticancer immunomodulators and the foundation for the next generation of cancer treatment modalities.
